# Supplementary material for: DeepSARS: simultaneous diagnostic detection and genomic surveillance of SARS-CoV-2
Source: BMC Genomics. 2022 Apr 11;23:289. doi: 10.1186/s12864-022-08403-0 (PMC8995413; doi:10.1186/s12864-022-08403-0)
Supplement: Supplementary file 1 — Additional file 1: Figure S1. Heatmap demonstrating the number of mutations separating the patient barcodes. The rows/columns correspond to a distinct 10 nucleotide barcode sequence. Intensity corresponds to the number of mutations necessary to convert one barcode into another. Diagonal corresponds to the distance between identical barcodes (distance=0). Figure S2. Phylodynamic inference of effective reproduction number and pandemic origin of five different alignments using either sites covered by DeepSARS or all sites in whole genome sequencing. Dashed line indicates the prior distribution. Figure S3. Experimental setting of the experiment depicted in Fig. 2C. Figure S4. Experimental setting of the experiment depicted in Fig. 2D. Figure S5. Logo plots depicting the per-base heterogeneity for the consensus sequences recovered from all reads in the experiment depicted in Fig. 2E. Boxes indicate those sites with defined mutations separating Twist synthetic RNA Controls 4 and 14. Letter size indicates the proportion of reads containing each specific nucleotide at the indicated base. Figure S6. Experimental setting of the experiment depicted in Fig. 3. Figure S7. The fraction of aligned reads on site-13 viral primers and two human gene-specific primers (RNAP and GAPDH) of COVID-19 patients and healthy controls for both swab and saliva samples. [file 12864_2022_8403_MOESM1_ESM.docx]

Supporting information:

**DeepSARS: simultaneous diagnostic detection and genomic surveillance of SARS-CoV-2**

Alexander Yermanos^1,2,3,4,^*^,#^, Kai-Lin Hong^1,2,^*, Andreas Agrafiotis^1,2,3^, Jiami Han^1,2^, Sarah Nadeau^1^, Cecilia Valenzuela^1^, Asli Azizoglu^1^, Roy Ehling^1^, Beichen Gao^1^, Michael Spahr^1^, Daniel Neumeier^1^, Ching-Hsiang Chang^1^, Andreas Dounas^5^, Ezequiel Petrillo^6,7^, Ina Nissen^1^, Elodie Burcklen^1^, Mirjam Feldkamp^1^, Christian Beisel^1^, Annette Oxenius^3^, Miodrag Savic^8^, Tanja Stadler^1^, Fabian Rudolf^1,#^, Sai T. Reddy^1,2,#^

^1^Department of Biosystems Science and Engineering, ETH Zurich, Basel, Switzerland. ^2^Botnar Research Centre for Child Health, Basel, Switzerland. ^3^Institute of Microbiology, ETH Zurich, Zurich, Switzerland. ^4^Department of Pathology and Immunology, University of Geneva, Geneva, Switzerland. ^5^Institute for Biomedical Engineering, University and ETH Zurich, Zurich, Switzerland. ^6^Instituto de Fisiología, Biología Molecular y Neurociencias (IFIBYNE-UBA-CONICET), Ciudad Universitaria, Buenos Aires, Argentina. ^7^Facultad de Ciencias Exactas y Naturales, Universidad de Buenos Aires, Ciudad Universitaria, Buenos Aires, Argentina. ^8^Department of Health, Economics and Health Directorate Canton Basel-Landschaft. *equal contribution. ^#^correspondence: [sai.reddy@ethz.ch](mailto:sai.reddy@ethz.ch), [fabian.rudolf@bag.admin.ch](mailto:fabian.rudolf@bag.admin.ch), ayermanos@gmail.com


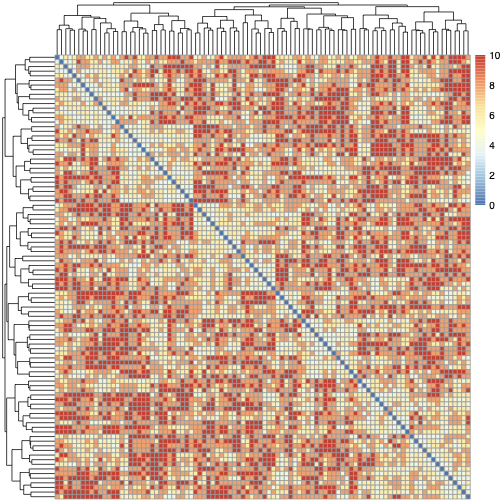


**Figure S1.** **Heatmap demonstrating the number of mutations separating the patient barcodes.** The rows/columns correspond to a distinct 10 nucleotide barcode sequence. Intensity corresponds to the number of mutations necessary to convert one barcode into another. Diagonal corresponds to the distance between identical barcodes (distance=0).


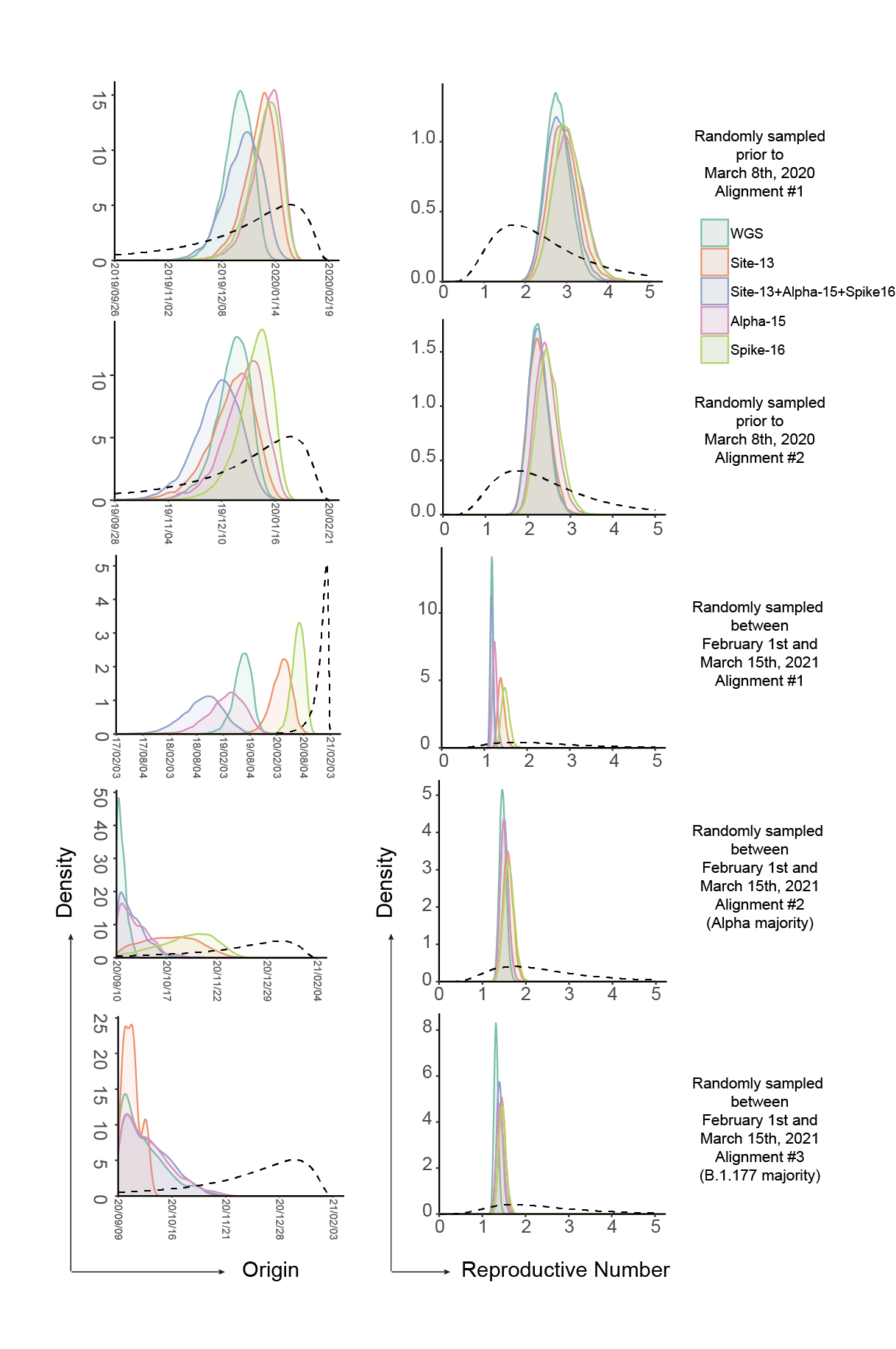


**Figure S2. Phylodynamic inference of effective reproduction number and pandemic origin of five different alignments using either sites covered by DeepSARS or all sites in whole genome sequencing.** Dashed line indicates the prior distribution.


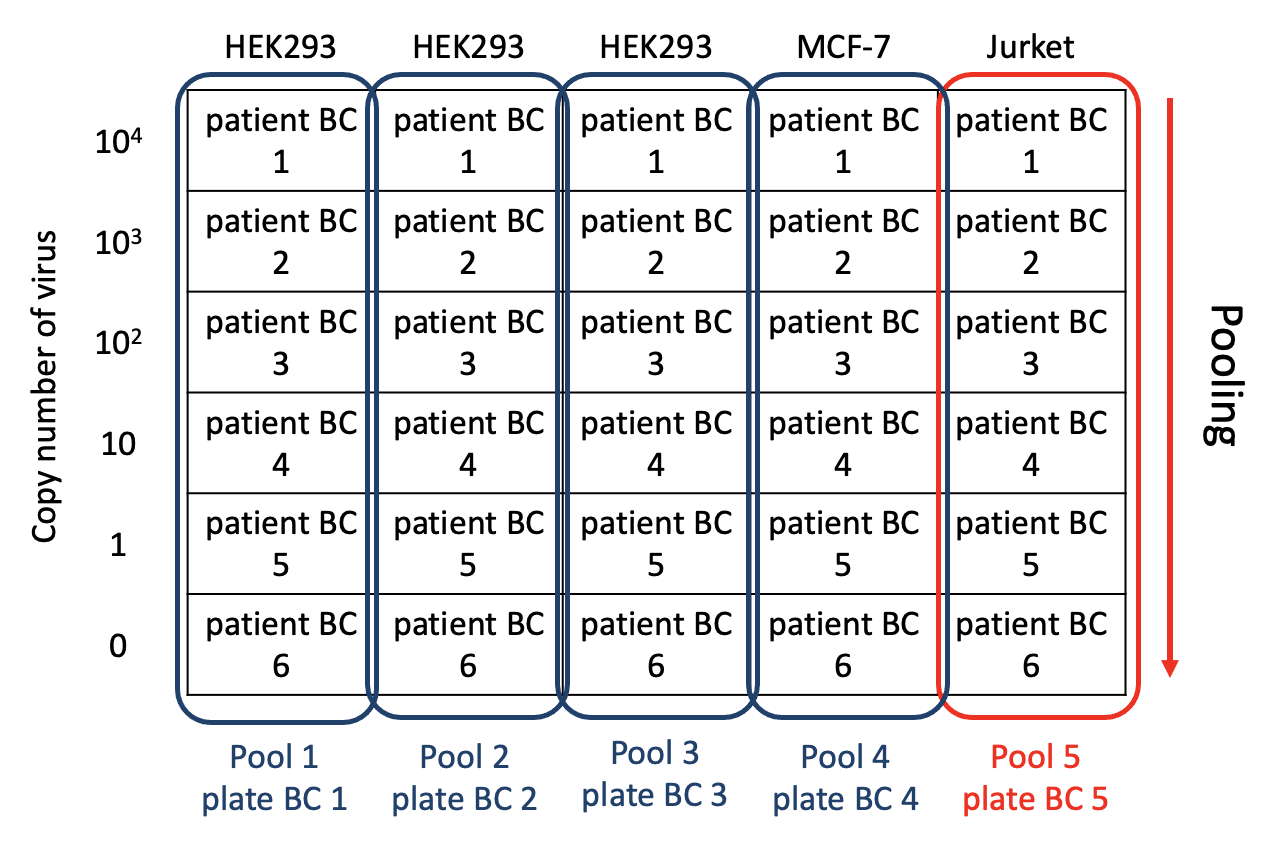


**Figure S3. Experimental setting of the experiment depicted in Figure 2C.**


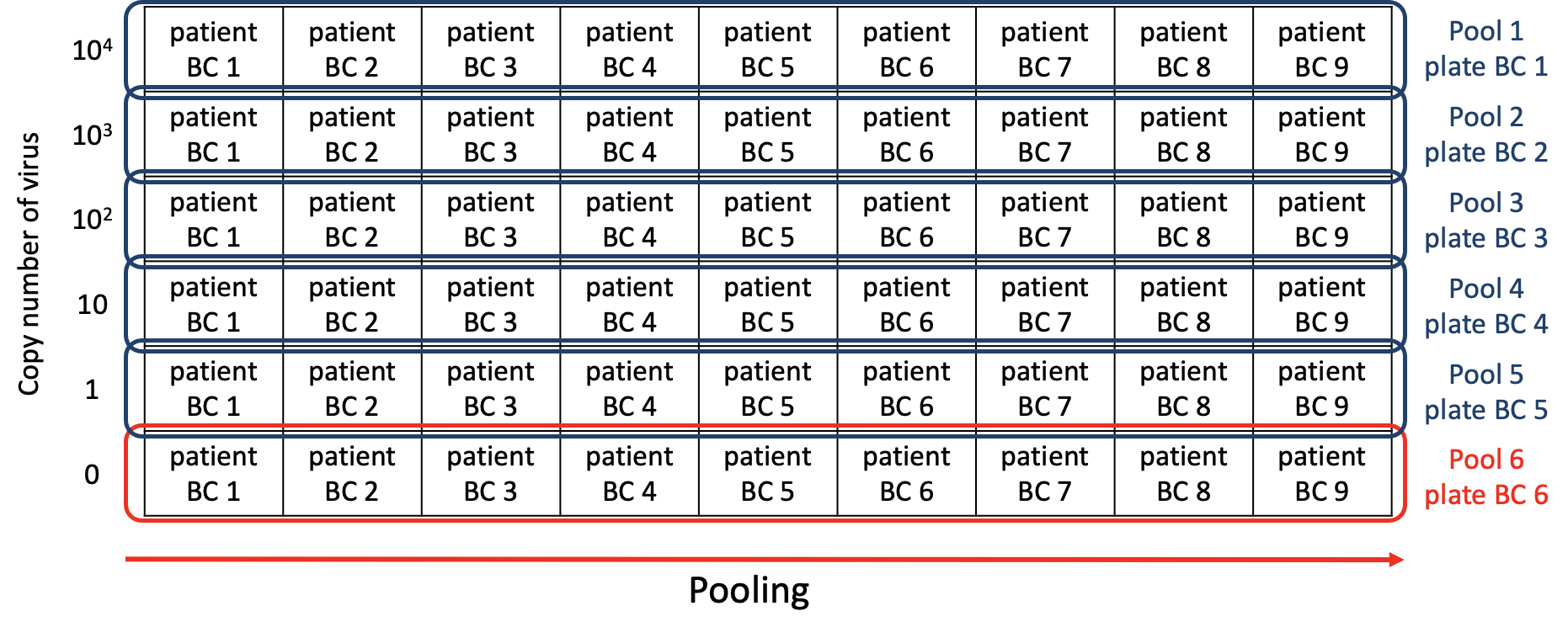


**Figure S4. Experimental setting of the experiment depicted in Figure 2D.**


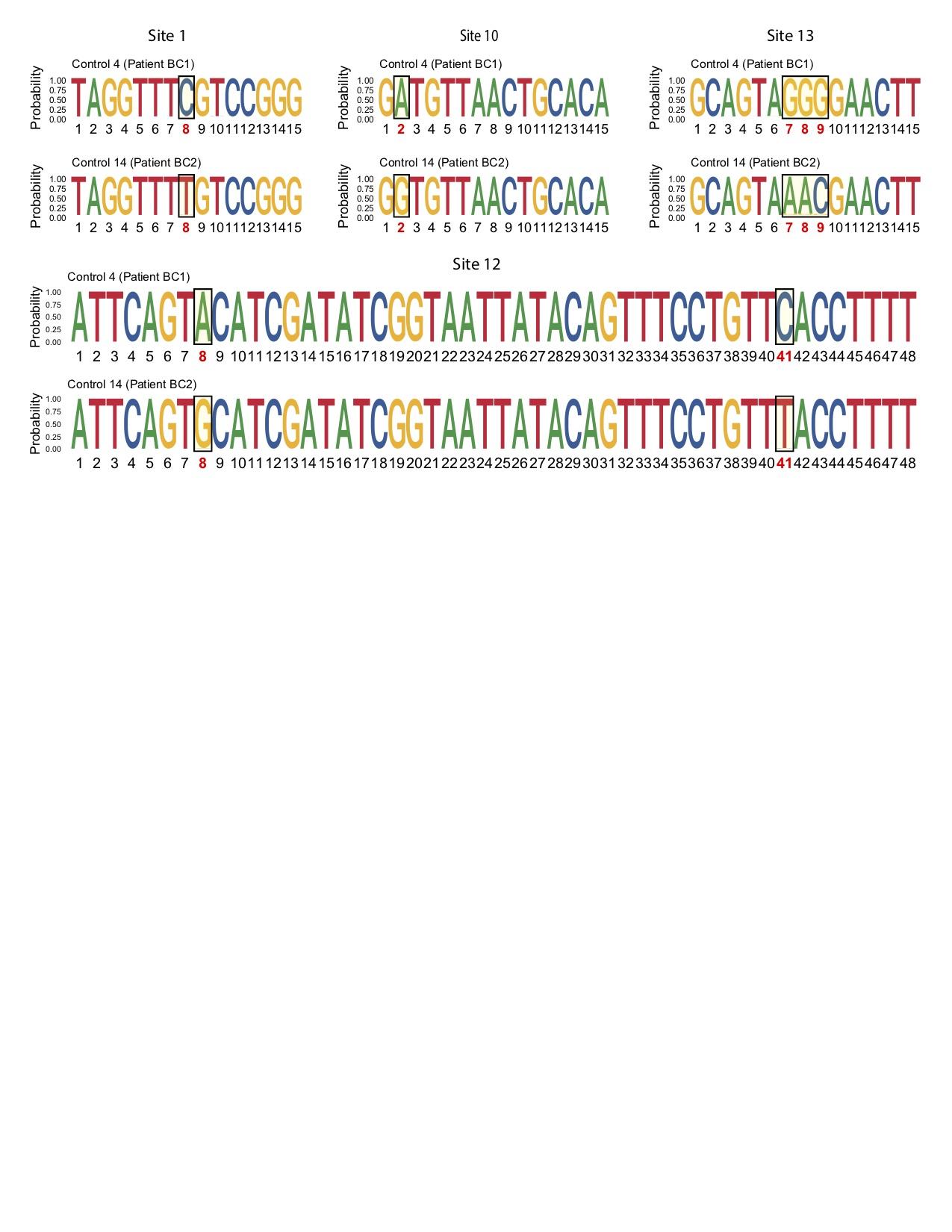


**Figure S5. Logo plots depicting the per-base heterogeneity for the** consensus sequences **recovered from all reads in the experiment depicted in Figure 2E.** Boxes indicate those sites with defined mutations separating Twist synthetic RNA Controls 4 and 14. Letter size indicates the proportion of reads containing each specific nucleotide at the indicated base.


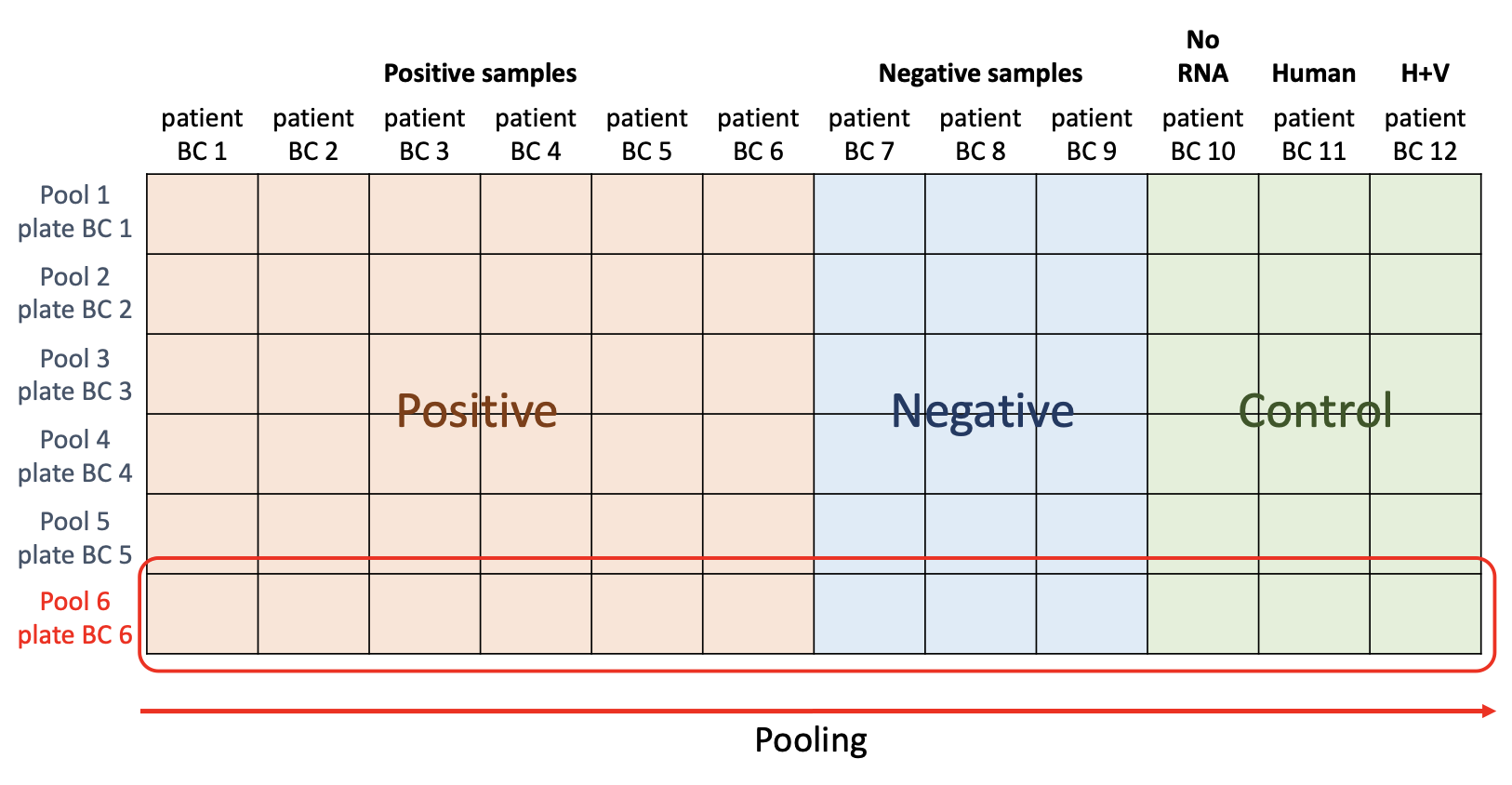


**Figure S6. Experimental setting of the experiment depicted in Figure 3.**

**Figure S7. The fraction of aligned reads on site-13 viral primers and two human gene-specific primers (RNAP and GAPDH) of COVID-19 patients and healthy controls for both swab and saliva samples.**
